# The characteristics and clinical relevance of tumor fusion burden in non-EBV (+) gastric cancer with MSS

**DOI:** 10.1186/s12876-023-02765-9

**Published:** 2023-05-15

**Authors:** Yongjun Zhu, Weixin Wu, Liangliang Qiao, Jingfen Ji, Lunxi Duan, Longlong Gong, Dandan Ren, Feifei Li, Lihui Wei, Ke Pan

**Affiliations:** 1 Department of Gastrointestinal surgery, The Second Clinical Medical College, The First Affiliated Hospital, Shenzhen People’s Hospital, Jinan University, Southern University of Science and Technology), Shenzhen, 518020 China; 2 Department of Oncology, Zhongshan Hospital affiliated to Xiamen University, Xiamen, 361004 China; 3 Oncology Department I, Jinshazhou Hospital of Guangzhou University of Chinese Medicine, Guangzhou, 510000 China; 4 Department of General Surgery, The Second Xiangya Hospital of Central South University, 410011 Changsha, China; 5 Department of Medicine, Genecast Biotechnology Co., Ltd, 214000 Wuxi, China

**Keywords:** Gastric cancer, Tumor fusion burden, Immunogenicity, Prognosis

## Abstract

**Background:**

Next-generation sequencing (NGS) is maturely applied for gene fusion detection. Although tumor fusion burden (TFB) has been identified as an immune marker for cancer, the relationship between these fusions and the immunogenicity and molecular characteristics of gastric cancer (GC) patients remains unclear. GCs have different clinical significance depending on their subtypes, and thus, this study aimed to investigate the characteristics and clinical relevance of TFB in non-Epstein–Barr-virus-positive (EBV+) GC with microsatellite stability (MSS).

**Methods:**

A total of 319 GC patients from The Cancer Genome Atlas stomach adenocarcinoma (TCGA-STAD) and a cohort of 45-case from ENA (PRJEB25780) were included. The cohort characteristics and distribution of TFB among the patients were analyzed. Additionally, the correlations of TFB with mutation characteristics, pathway differences, relative abundance of immune cells, and prognosis were examined in the TCGA-STAD cohort of MSS and non-EBV (+) patients.

**Results:**

We observed that in the MSS and non-EBV (+) cohort, the TFB-low group exhibited significantly lower gene mutation frequency, gene copy number, loss of heterozygosity score, and tumor mutation burden than in the TFB-high group. Additionally, the TFB-low group exhibited a higher abundance of immune cells. Furthermore, the immune gene signatures were significantly upregulated in the TFB-low group, 2-year disease-specific survival was markedly increased in the TFB-low group compared with to the TFB-high group. The rates of TFB-low cases were significantly higher TFB-than high cases in durable clinical benefit (DCB) and response groups with pembrolizumab treatment. Low TFB may serve as a predictor of GC prognosis, and the TFB-low group exhibits higher immunogenicity.

**Conclusion:**

In conclusion, this study reveals that the TFB-based classification of GC patient may be instructive for individualized immunotherapy regimens.

**Supplementary Information:**

The online version contains supplementary material available at 10.1186/s12876-023-02765-9.

## Introduction

Gastric cancer (GC) is a malignant originating from the epithelium of the gastric mucosa with a progressively increasing mortality rate in recent years [1]. Although improved surveillance technologies have facilitated early detection and intervention, most GC cases are diagnosed at an advanced stage, which limits therapeutic options and results in poor 5-year survival rates [2]. Currently, surgical resection in combination with neoadjuvant or adjuvant chemotherapy is the mainstay of treatment for GC [3]. However, due to the heterogeneity of GC, including the Lauren and WHO histological classifications, treatment responses and efficacy may vary greatly between patients. With the advent of next-generation sequencing (NGS), GC in The Cancer Genome Atlas (TCGA) has been categorized into four subtypes: Epstein–Barr-Virus positivity (EBV+), microsatellite instability (MSI), genome stable (GS), and chromosomal instability [4]. These classifications have important biological and clinical significance for the diagnosis, treatment, and basic research of GC. Recent studies have found that GC patients with MSI and EBV (+) are more likely to benefit from immunotherapy [5, 6]. Nevertheless, most patients will continue to experience progression-free survival and there are still no specific markers related to the prognosis and treatment of GC patients with microsatellite stable (MSS) and non-EBV (+).

A fusion gene arises from the integration of two or more independent genes via intra-chromosomal or inter-chromosomal recombination [7]. Previous investigations using NGS data from various cancer types have revealed multiple gene fusions [8]. Tumor fusion burden (TFB) is defined as the number of fusion genes per 10,000 genes and many fusion genes that can be used as molecular diagnostic markers have been found in soft tissue tumors [9], epithelial cancer [10], and prostate cancer [11]. Moreover, fusion genes that are active or promote abnormally high expression of the original gene have been determined in GC research, which is also a potential driver in the development and progression of GC [12]. Consequently, an in-depth study of fusion gene characteristics holds significant clinical importance. Furthermore, gene fusions are also associated with the immune status of the tumor microenvironment (TME) [7]. A previous study highlighted a negative relationship between gene fusions and cytolytic immune signatures (such as NK cells and CD8 + T cells) across 85 prostate cancers [13]. Recent findings also suggest that TFB can function as an immune marker for prostate cancer [11]. However, the correlation of TFB with tumor immunogenicity and molecular characteristics in GC remains to be fully investigated.

This study analyzed data from the TCGA-stomach adenocarcinoma (STAD) cohort and divided GC patients into the TFB-high and TFB-low groups according to the upper quartile of TFB. Furthermore, we performed comparative studies on gene mutation characteristics and immunological and clinical characteristics of patients between the two groups, as well as a comprehensive assessment of the correlation between fusion load and tumor immunity in the cohort.

## Results

### Characteristics and TFB distribution of GC patient in the TCGA-STAD cohort

**Fig. 1:**
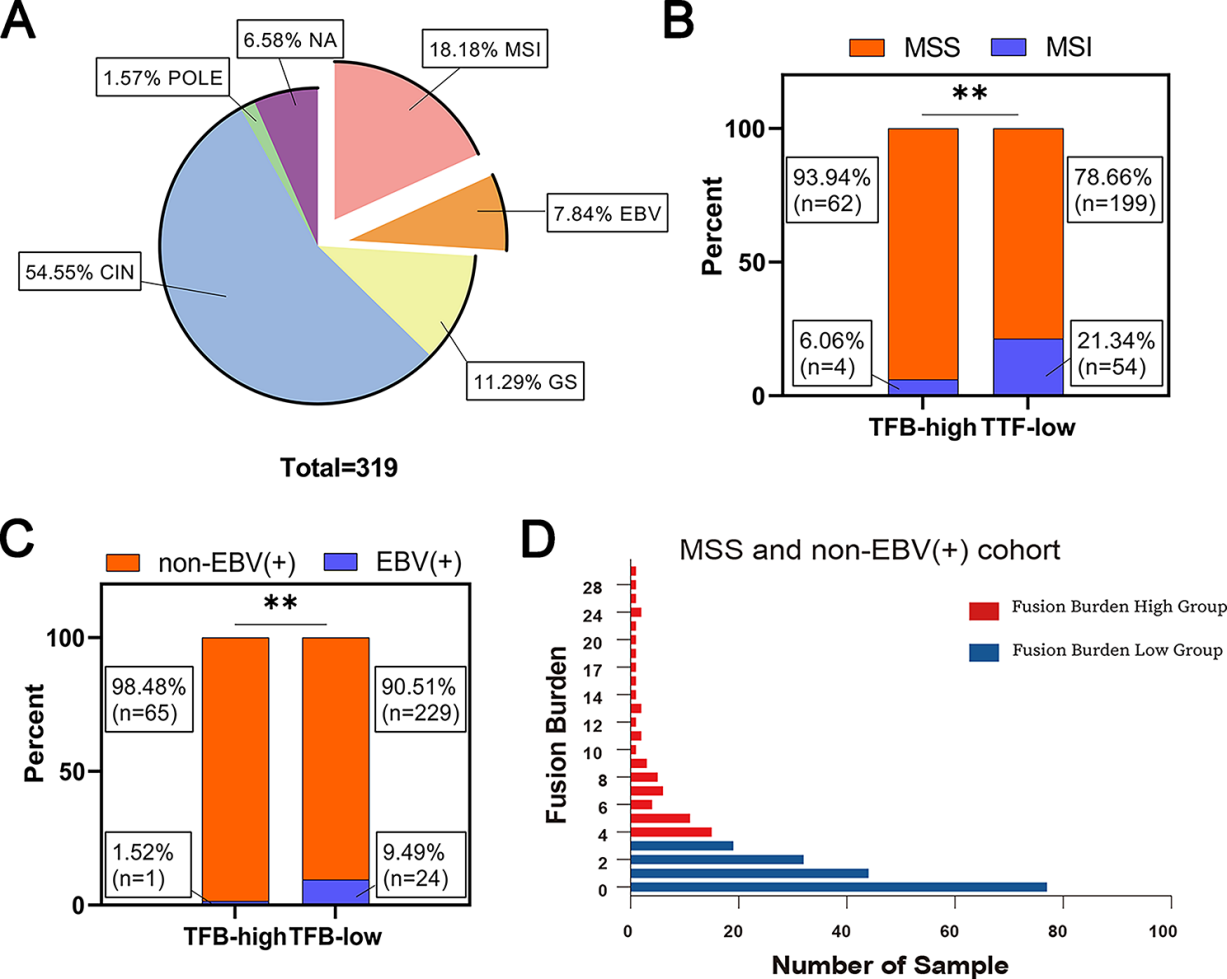
Characteristics of the TCGA-STAD cohort and TFB distribution. (**A**) Type distribution in the cohort; (**B**) Relationship of microsatellite instability with MSS and high/low TFB group; (**C**) Relationship between EBV (+) status and high/low TFB group; (**D**) TFB distribution in the MSS and non-EBV (+) cases. Data were analyzed by Fisher’s exact test (two sided). **p < 0.01

The TCGA-STAD cohort included 319 patients with gastric adenocarcinoma, of which MSI and EBV (+) cases accounted for 18.18% and 7.84%, respectively (Fig. 1A), and the rest was 73.98%, the majority. Comparative analysis revealed a significantly higher proportion of MSI and EBV(+) cases in the TFB-low group compared to the TFB-high group (*p*<0.01), whereas the proportion of MSS and non-EBV (+) cases was significantly lower in the TFB-low group (*p* < 0.01) (Fig. 1B C). Based on these findings, subsequent studies were performed on the MSS and non-EBV (+) cohort. As shown in Fig. 1D, TFB values in the MSS and non-EBV (+) cases ranged from 0 to 29. TFB > 3 was considered TFB high (61 cases) and the remaining was considered TFB low (172 cases). The characteristics of this cohort were presented in Table S1.

### Mutational landscape of patients with high or low TFB in the MSS and non-EBV (+) cohort

**Fig. 2:**
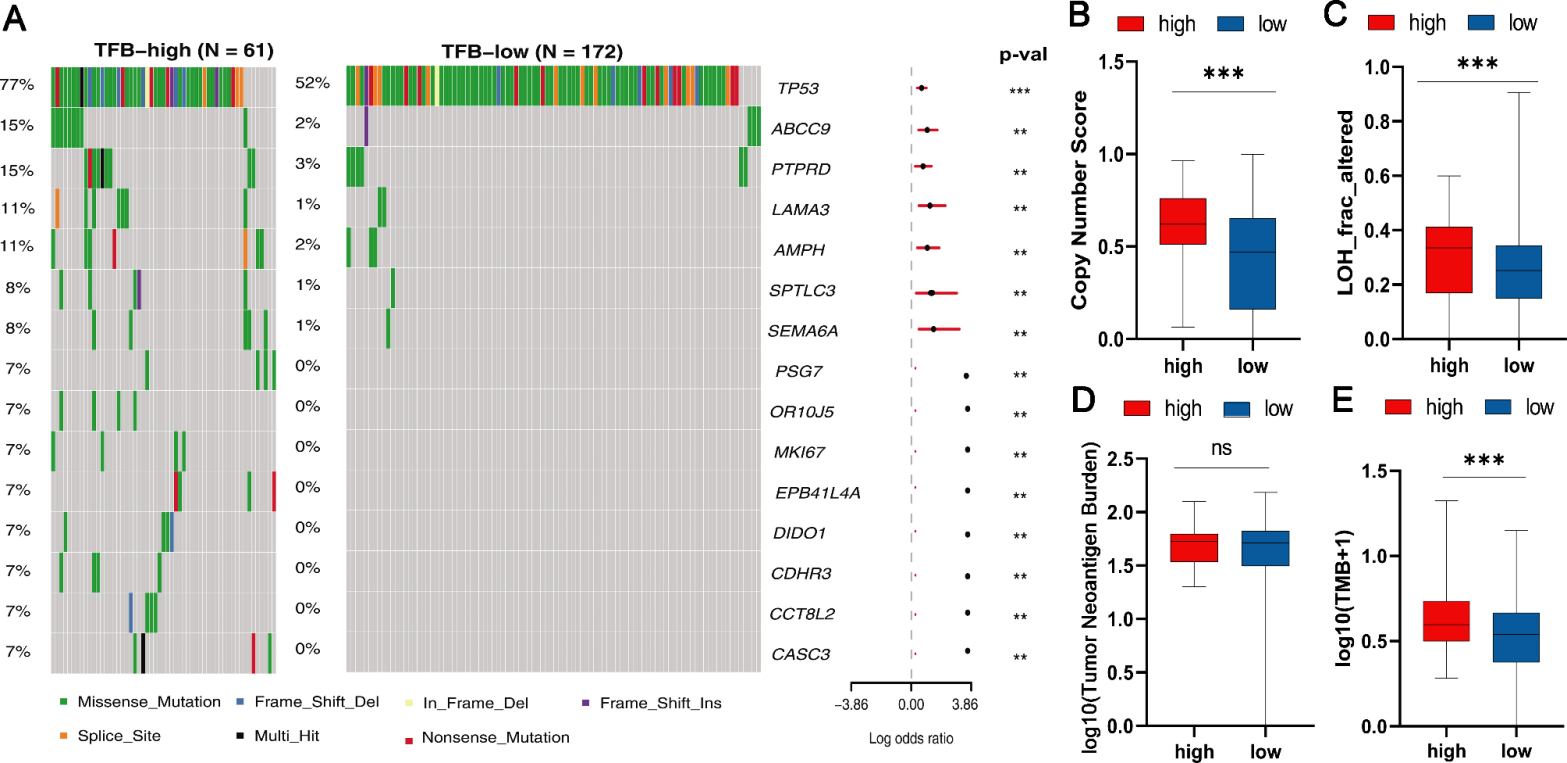
Mutational landscape and genomic pattern of patients with high or low TFB in the MSS and non-EBV (+) cohort. (**A**) The mutation spectrum of high/low TFB groups; (**B**-**E**) Comparisons of copy number score (**B**), LOH fraction (**C**), tumor neoantigen burden (**D**), and tumor mutation burden (**E**) between TFB-high and TFB-low groups. Data were analyzed by Wilcox test. Significant differences: *p < 0.05, **p < 0.01, ***p < 0.001. No significant difference is indicated as ns

Next, we analyzed the gene mutation landscape in the TFB high and low groups to determine whether TFB was linked to other mutation types. As depicted in Fig. 2A, the TP53 gene was found to be the most frequently mutated one in the MSS and non-EBV (+) cohort, followed by ABCC9, PTPRD, LAMA3, AMPH, and other genes as indicated. Furthermore, the mutation frequencies of all these genes were significantly lower in the TFB Low group compared to the TFB high group (*p* < 0.001). We also observed that the gene copy number, LOH score, and TMB value in the TFB Low group were significantly lower than that in the TFB high group (*p* < 0.001) (Fig. 2B-C and E), while there was no significant difference in the number of immunogenic mutations between the two groups (Fig. 2D).

### Tumor immune activity of patients with high or low TFB in the MSS and non-EBV (+) cohort

**Fig. 3:**
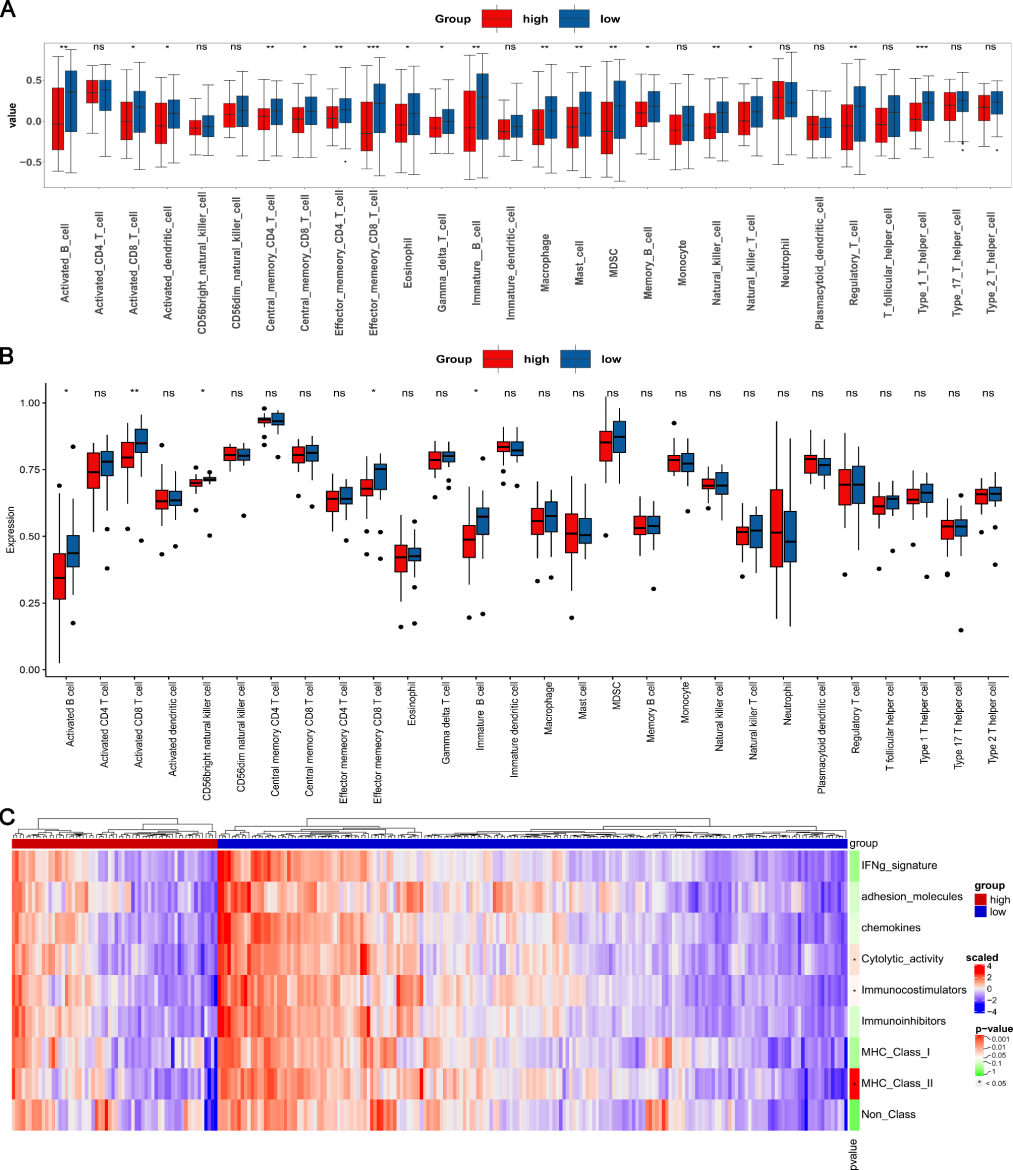
Tumor immune activity of patients with high or low TFB in the MSS and non-EBV (+) cohort. (**A** and **B**) The abundance of 28 immune cell subpopulations in the TGCA GC cohort and the 45-case cohort from ENA (PRJEB25780). Data were analyzed by Wilcoxon test. Significant differences: *p < 0.05, **p < 0.01, and ***p < 0.001. No significant difference is indicated as ns. (**C**) The immune gene signature. Data were analyzed by Wilcox test. *p < 0.05

We comparatively analyzed the immune cell infiltration between the two groups by calculating the score of 28 immune cell infiltration using ssGSEA. As shown in Fig. 3A, compared with the TFB-high group, several immune cells were more enriched in the TFB-low group (*p* < 0.05) from the TCGA GC cohort, including activated B cells, activated CD8 T cells, activated dendritic cells, central memory CD4 T cells, central memory CD8 T cells, effector memory CD4 T cells, effector memory CD8 T cells, eosinophils, gamma-delta T cells, immature B cells, macrophages, mast cells, MDSCs, memory B cells, natural killer cells, natural killer T cells, regulatory T cells, and type 1 T helper cells. In the 45-case cohort from ENA (PRJEB25780), we also found that many immunity cells such as activated B cells, activated CD8 T cells, effector memory CD8 T cells, immature B cells, were more in the TFB-low cases than the high group (Fig. 3B). Similarly, the immune signatures such as cytolytic activity, immune co-stimulators, and MHC Class II were significantly enhanced in the TFB-low group (*p* < 0.05) (Fig. 3C).

### Pathway analysis of patients with high or low TFB in the MSS and non-EBV (+) cohort

**Fig. 4:**
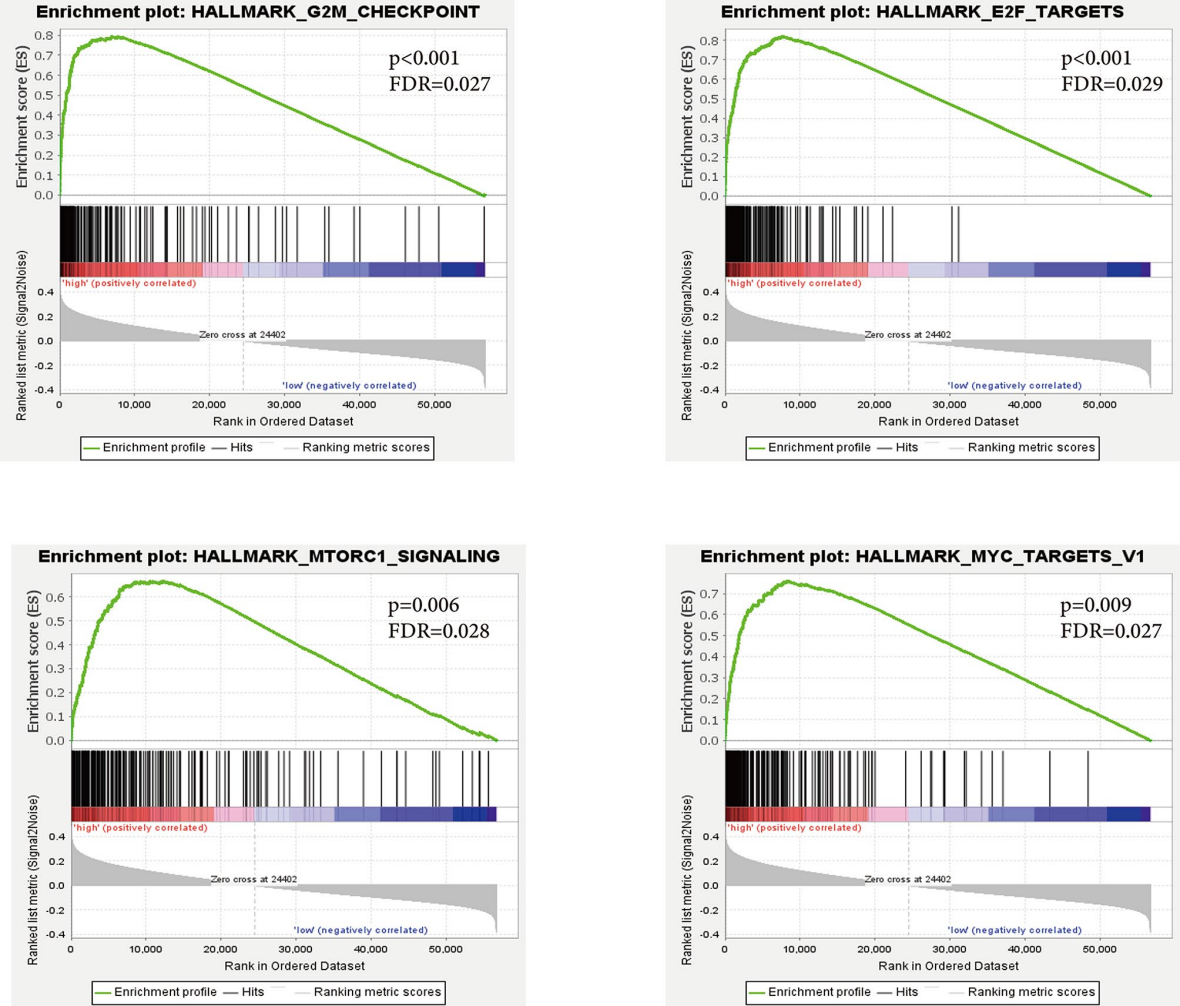
GSEA-based pathway enrichment analysis of patients with high or low TFB in the MSS and non-EBV (+) cohort

**Fig. 5:**
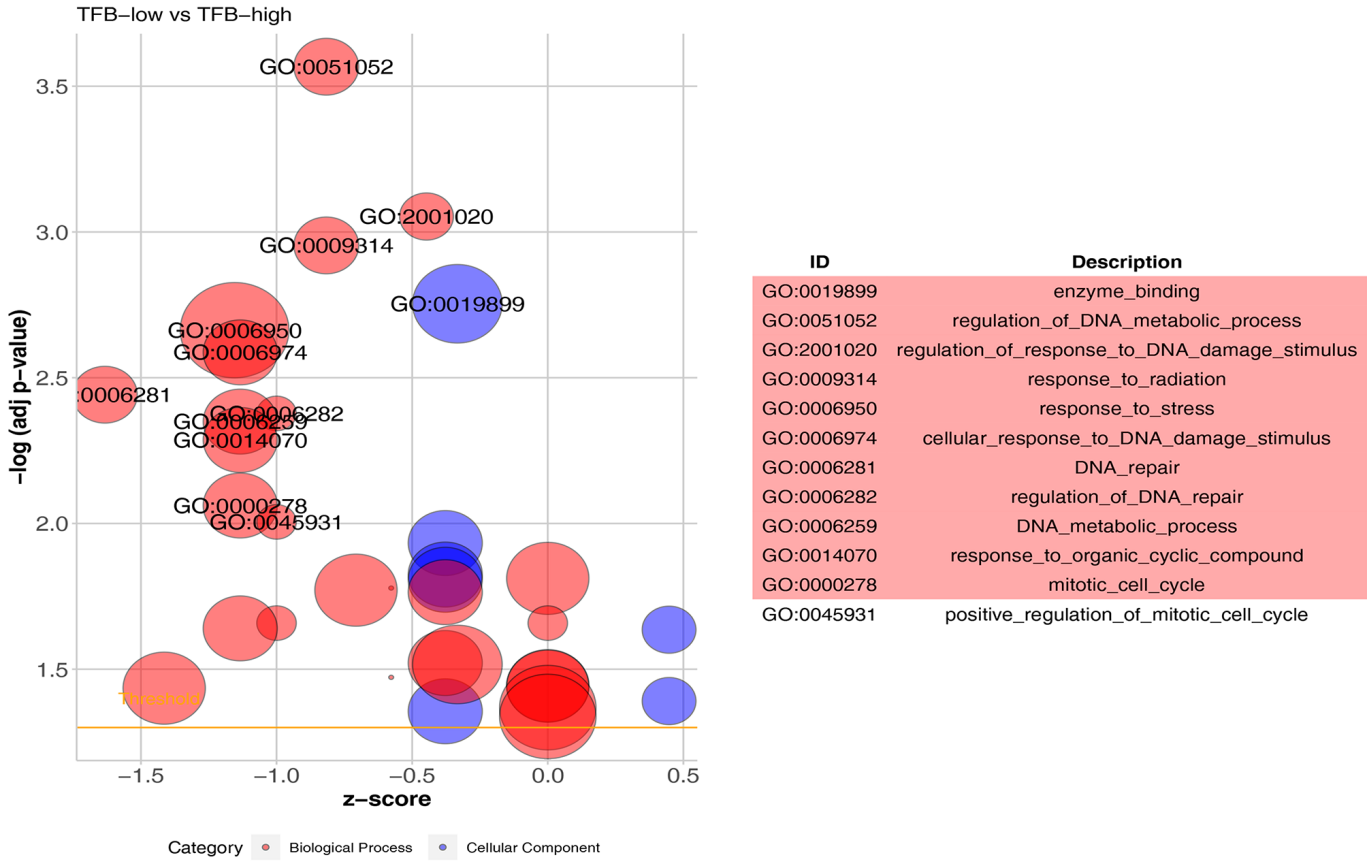
GO enrichment analysis of patients with high or low TFB in the MSS non-EBV (+) cohort

Then, we further performed GSEA and GO analysis to explore pathways associated with TFB differences in the TCGA GC cohort. As illustrated in Fig. 4, high TFB was positively correlated with the enrichment of multiple pathways, such as G2M checkpoint (*p* < 0.001, *FDR* = 0.027), E2F targets (*p* < 0.001, *FDR* = 0.029), MTORC1 signaling (*p* = 0.006, *FDR* = 0.028), and MYC targets (*p* = 0.009, *FDR* = 0.027). Meanwhile, GO enrichment analysis revealed that several pathways, including DNA repair, were down-regulated in the TFB-low group (*Z-score* < 0) (Fig. 5).

### Prognosis of GC patients with high or low TFB

**Fig. 6:**
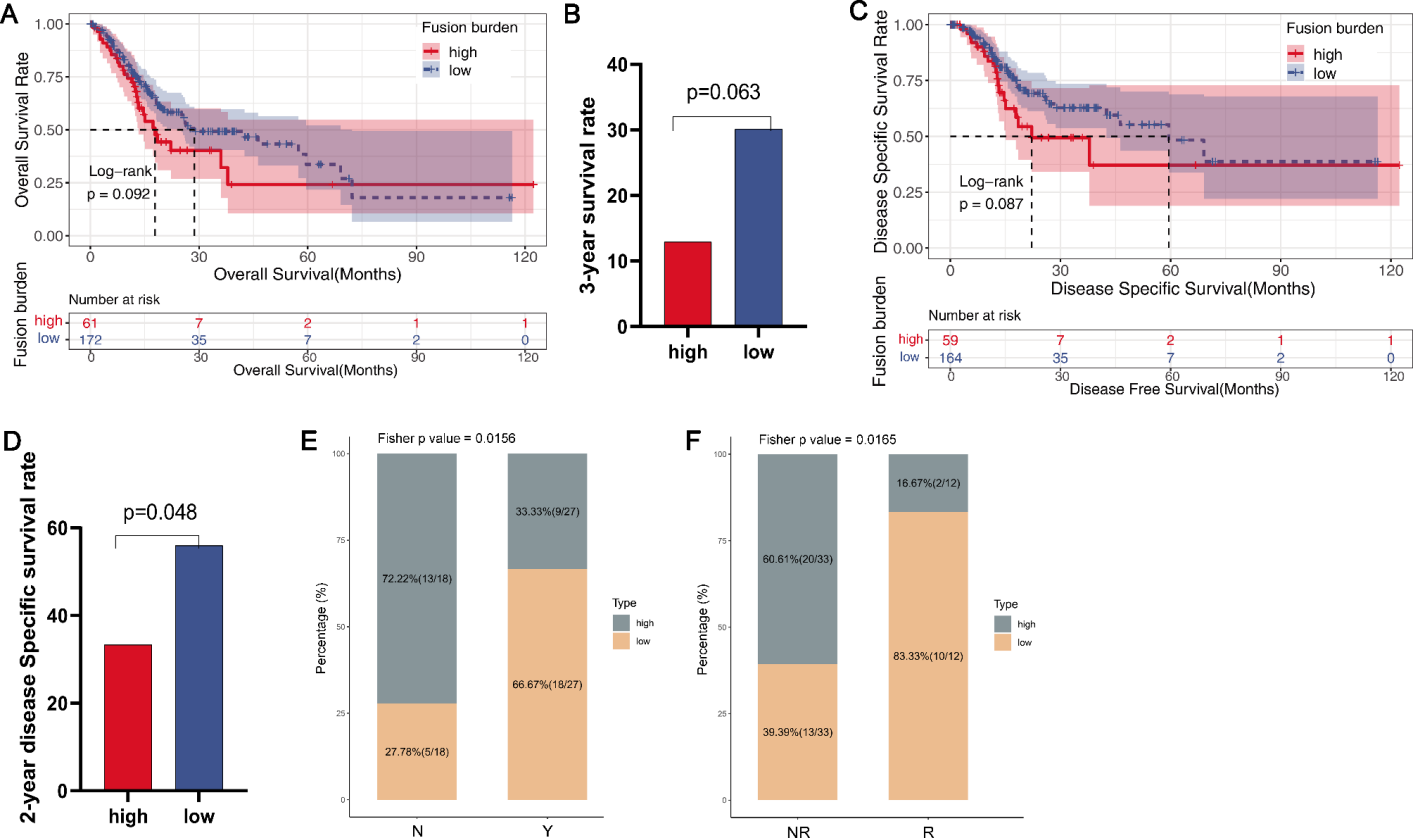
Survival and response analysis of GC patients with high or low TFB. (**A** and **B**) Differences in the overall survival (**A**) and 3-year survival rate (**B**) between the groups were analyzed by log-rank and Fisher’s exact test (two sided), respectively. (**C** and **D**) Differences in disease-specific survival time (**C**) and 2-year disease Specific survival rate (**D**) between the groups were analyzed by log-rank and Fisher’s exact test (two sided), respectively. (**E** and **F**) Durable clinical benefit (DCB; **E**) and response (**F**) analysis with pembrolizumab treatment in the 45 GC patients from ENA (PRJEB25780). Data were analyzed by Fisher test. Significant differences: *p* < 0.05

As shown in Fig. 6A-D, the TFB-low group represented an increase in overall survival (log-rank, *p* = 0.092), 3-year survival rate (Fisher, *p* = 0.063), disease-specific survival (log-rank, *p* = 0.087), and 2-year disease-specific survival (Fisher, *p* = 0.048) compared to the TFB-high group, albeit there were no significant differences in most prognostic indicators between the two groups. But in the 45-case cohort from ENA (PRJEB25780) with immunotherapy, the results showed that rates of the TFB-low cases were significantly more than the high cases in durable clinical benefit (DCB; Fisher, *p* = 0.0156) and response (Fisher, *p* = 0.0165) groups with pembrolizumab treatment (Fig. 6E F). These results indicated that TFB might serve as a predictor of prognosis in GC patients with immunotherapy.

## Discussion

GC is a prevalent malignancy worldwide, with a complex pathogenic mechanism involving multiple factors, such as genetics, environment, and epigenetics [2]. Gene fusions are common cancer-causing mutations and play significant roles in the initial steps of tumorigenesis [14]. In the present study, we illuminated that GC patients in the TFB-low group had a relatively high incidence of EBV infection and MSI. While EBV (+) and MSI GC have been studied extensively as independent subtypes of GC due to their distinctive clinicopathological and molecular biological characteristics [15, 16], there is a lack of relevant reports for the majority of GC patients who do not belong to these subtypes. Therefore, this study aimed to explore the impact of TFB on the clinicopathological and molecular characteristics of MSS and non-EBV (+) GC patients. Result showed that the TFB-low group had lower gene mutation frequency, Copy Number Score, LOH, and TMB, and higher immunogenicity. Additionally, high TFB was positively correlated with the enrichment of the E2F pathway, while several pathways including DNA repair were down-regulated in the TFB-low group. Meanwhile, the 2-year disease-specific survival was increased in the TFB-low group compared to the TFB-high group. We further validated these findings in a cohort of 45 cases treated with ENA (PRJEB25780) immunotherapy, demonstrating that cases with low TFB had a significantly higher clinical benefit and treatment response than those with high TFB. These findings provides novel insights into the pathogenesis and management of non-EBV (+) and MSS GC patients. Our findings suggest that TFB expression can serve as a potential prognostic biomarker and a therapeutic target for GC.

Research pointed out that fusion genes have been found to commonly coexist with gene mutations and play a role in GC progression [12]. Our findings suggest that patients with high TFB have higher mutation rates, especially in genes such as TP53, ABCC9, PTPRD, LAMA3. This observation is consistent with the previous report that the random frequency of concomitant fusion genes and TP53 mutations occurring in tumors is higher than expected [17]. Similarly, in patients with T-cell acute lymphoblastic leukemia, fusions were often accompanied by at least one C1–C7 mutation, revealing possible cooperative effects [18]. Moreover, patients with high TFB displayed higher gene copy numbers, LOH scores, and TMB values, indicating that gene fusions and mutations in tumors are consistent [17]. Paradoxically, fusion-negative patients with rhabdomyosarcoma (RMS) harbored more genomic alterations and higher TMB values than fusion-positive cases [19]. Conversely, in ALK fusion-negative NSCLC patients, fusion-positive cases exhibited a relatively lower frequency of TP53 mutation, lower TMB values, and fewer co-mutations [20]. These findings have implications for the treatment of fusion gene-related cancers.

As a heterogeneous disease, GC displays unique immunological characteristics, and TME has been shown to play a vital role in the progression of GC. To characterize the association between TFB and TME, we evaluated the relative abundance of 28 infiltrating immune cells in the TCGA-STAD MSS non-EBV (+) cohort. Our findings revealed that B cells and CD8 T cells were more enriched in the TFB-low group. Interestingly, gene fusion was negatively correlated with cellular immune characteristics in prostate cancer patients [13], and we found that the TFB-low group expressed higher levels of cytolytic activity, immune co-stimulators, and MHC Class II. This suggests that TFB-low patients may benefit more from immunotherapy than TFB-high ones. An earlier study demonstrated that the cytolytic activity score reflects anti-tumor immunity and is related to the clinical outcome of GC patients [21]. And the host immune system can recognize MHC-II positive tumor cells and develop a protective immune response [22]. Additionally, MHC II has been shown to activate tumor-specific CD4 + and CD8 + T cells, promoting immunogenicity in uveal melanoma [23]. It has been reported that the formation of an immunosuppressive microenvironment normally prevents tumor killer cells from the clearance of tumor cells, leading to an increased risk of malignant progression and death [24]. Therefore, targeting the immunosuppressive microenvironment may be a more effective and feasible treatment strategy for patients with low TFB in the MSS and non-EBV (+) GC.

Additionally, enrichment analysis revealed high TFB enrichment in pathways associated with cancer development, providing a possible mechanism for TFB relevance. Research has suggested that the E2F pathway reflects tumor aggressiveness and responsiveness to therapy [25]. In tumors with high E2F pathway activity, several cell proliferation-related gene sets are highly expressed, including G2M checkpoints, MTORC1 signaling, and MYC targets. Reports have also shown that a high E2F pathway score is related to worse clinical features [25]. Given that tumors with gene fusions tend to exhibit high malignancy and rapid progression, we hypothesized that a higher E2F pathway score would be linked to gene fusions. Indeed, we found that high TFB activates G2M checkpoints, E2F targets, MTORC1 signaling, and MYC targets, facilitating tumor occurrence and development. Additionally, GO enrichment analysis revealed that several pathways, including DNA repair, were down-regulated in the TFB-low group. DNA repair plays a crucial role in the response to human cancer treatment [26], and assessing DNA repair pathways may help identify cancer patients with higher immunogenicity who may have a favorable response to immunotherapy [27]. As GC is a common cancer with poor prognosis [21], determining the predictors of GC prognosis is crucial. Also, the prognosis of GC patients is associated with gene fusion. In the TCGA study, the CLDN18-ARHGAP26 fusion gene was found to be existed in GS/diffuse GC [12], and it could promote epithelial-mesenchymal transition and enhance the invasion and metastasis ability of tumor cells, thereby affecting the prognosis of patients. In addition, in hormone receptor-positive (HR+) breast cancer, patients with positive rearrangement-mediated expression of gene fusions have a lower overall survival rate than those with negative gene fusions[28]. Consistent with this, we found that high TFB was related to poor survival status and relapse in GC, with increased 2-year disease-specific survival in the TFB-low group compared to the TFB-high group, demonstrating that low TFB may be used to predict the prognosis of GC patients, as well as being a routine parameter for the selection of immunotherapy in those patients.

## Conclusion

In summary, the identification of low TFB as a potential predictor of GC prognosis is a significant finding with potential clinical implications. And GC patients with low TFB have higher immunogenicity and may be sensitive to immunotherapy. TFB-based GC classification could help clinicians to identify advantageous treatment populations and develop individualized immunotherapy regimens. At the same time, more clinical studies are needed to assess and verify the clinical potential of this GC classification.

## Materials and methods

### Data collection

Gene expression profiles and clinical data of GC patients were downloaded from the TCGA (TCGA, PanCancer Atlas) via the cBioPortal (http://www.cbioportal.org/). RNA sequencing data (FPKM) values were transformed into transcript per kilobase million (TPM) values.

### Tumor fusion burden (TFB) calculation

The TFB was defined as the number of fusion genes per 10,000 genes. Fusion data of GC released by TCGA published studies [29] were collected, which included 355 samples, of which 319 were tumor samples that were included in the study. Patients were categorized into the TFB-high or TFB-low group based on TFB = 3 in the TCGA GC cohort. TFB > 3 was considered as TFB high, while the remaining values were considered TFB low. Detailed information on TFB was shown in Supplementary data 1.xlsx. A 45 GC-case cohort with pembrolizumab treatment was obtained and screened from ENA (PRJEB25780) [30]. TFB was calculated using the RNA sequencing dataset (Supplementary data 2) of the GC cases. The low/high TFB groups was determined using the optimal Youden index. Detailed information on TFB was shown in Supplementary data 3.xlsx.

### GC molecular subtypes

GC cases from the TCGA cohort were divided into different molecular subtypes based on major pathogenic pathways (EBV-positive, MSI, GS, and CIN tumors). The simplified dichotomy algorithm was used to analyze EBV (+) status as described previously [31]. Based on the simplified dichotomy algorithm, an approximated reproduction of the TCGA classification was achieved by an algorithm that started with the analysis of the EBV positivity and then investigated the MSI status. Finally, the GS and CIN tumors were separated by the histological subtypes according to the Lauren classification (GS: diffuse, CIN: intestinal type).

### Gene variation feature analysis

The gene sets for cytolytic activity, IFN γ signature, immunocostimulators, immunoinhibitors, chemokines, and MHC-class-I/II signature were defined in previous reports [32, 33]. The immune gene signatures were measured as the mean value of gene expression in log2 of TPM. Tumor mutation burden (TMB), microsatellite instability (MSI), immunogenic mutation, copy number, and loss of heterozygosity (LOH) data were collected from published studies [34–38] on the TCGA cohort. The TMB, MSI, tumor neoantigen burden, copy number score, and LOH data were shown in Supplementary data 4.xlsx.

### Immunity signature analysis

The enrichment level of gene sets in each GC sample was quantified by the single-sample gene set enrichment analysis (ssGSEA) using a total of 28 immune marker gene sets defined by immune genome functions [39]. The expression of 28 immune cell types in the TCGA GC cohort and the 45-GC cohort were calculated using a GSVA package in R software version 3.6.1.

### Function and pathway analysis

The R package GOplot for gene ontology (GO) analysis was conducted to analyze the possible functions of these TFB differences, and then the GO pathway enrichment is performed (https://biit.cs.ut.ee/gprofiler/) [40]. The GSEA was performed to identify the differential regulatory pathways between the TFB-high and TFB-low groups [41]. GSEA (http://software.broadinstitute.org/gsea/index. jsp) was conducted based on the expression results using the default parameters on c2 gene sets in the Molecular Signatures Database (MSigDB) (http://software.broadinstitute.org/gsea/msigdb). Enriched pathways with a normal *p* value < 0.05 and a false discovery rate (FDR) Q value < 0.25 were considered statistically significant.

### Statistical analysis

The statistical analysis was performed using R software version 3.6.1 (http://www.R-project.org) and its corresponding packages and SPSS 22.0 version. The overall survival (OS) and disease-specific survival rate (DSS) were assessed using the Kaplan-Meier curve and tested via the log-rank method. Comparisons between the TFB-high and TFB-low groups were conducted by the Wilcox test or Fisher’s exact test (two-sided), and *p* < 0.05 was considered statistical significance.

## Electronic supplementary material

Below is the link to the electronic supplementary material.

Supplementary Material 1

Supplementary Material 2

Supplementary Material 3

Supplementary Material 4

Supplementary Material 5

## Data Availability

All the data in the study are included in the article/Supplementary Materials. Further inquiries can be directed to the corresponding authors.
