# Supplementary material for: The characteristics and clinical relevance of tumor fusion burden in non-EBV (+) gastric cancer with MSS
Source: BMC Gastroenterol. 2023 May 15;23:153. doi: 10.1186/s12876-023-02765-9 (PMC10184333; doi:10.1186/s12876-023-02765-9)
Supplement: Supplementary file 1 — Supplementary Material 1 [file 12876_2023_2765_MOESM1_ESM.doc]

**Table S1 The Clinical fetures between fusion burden high and low group in TCGA STAD MSS non-EBV (+) cohort (n=233)**

| **Factor** | **Charactersictic** | **Overall**  **(n=233)** | **TFB-high**  **(n=61)** | **TFB-low**  **(n=172)** | **P value** |
| --- | --- | --- | --- | --- | --- |
| AGE (median [IQR]) |  | 66.00 [57.50, 72.00] | 66.00 [58.00, 72.00] | 65.50 [57.25, 71.00] | 0.694 |
| Sex (%) | Female | 71 (30.5) | 21 (34.4) | 50 (29.1) | 0.536 |
|  | Male | 162 (69.5) | 40 (65.6) | 122 (70.9) |  |
| Satge (%) | NA | 3 (1.3) | 0 (0.0) | 3 (1.7) | 0.283 |
|  | Stage I | 31 (13.3) | 8 (13.1) | 23 (13.4) |  |
|  | Stage II | 75 (32.2) | 14 (23.0) | 61 (35.5) |  |
|  | Stage III | 102 (43.8) | 32 (52.5) | 70 (40.7) |  |
|  | Stage IV | 22 (9.4) | 7 (11.5) | 15 (8.7) |  |
| Ethnicity (%) | NA | 58 (24.9) | 17 (27.9) | 41 (23.8) | 0.698 |
|  | Hispanic Or Latino | 1 (0.4) | 0 (0.0) | 1 (0.6) |  |
|  | Not Hispanic Or Latino | 174 (74.7) | 44 (72.1) | 130 (75.6) |  |
| New tumor event after initial treatment (%) | NA | 22 (9.4) | 6 (9.8) | 16 (9.3) | 0.84 |
|  | No | 143 (61.4) | 39 (63.9) | 104 (60.5) |  |
|  | Yes | 68 (29.2) | 16 (26.2) | 52 (30.2) |  |
| M Stage (%) | M0 | 208 (89.3) | 52 (85.2) | 156 (90.7) | 0.316 |
|  | M1 | 14 (6.0) | 4 (6.6) | 10 (5.8) |  |
|  | MX | 11 (4.7) | 5 (8.2) | 6 (3.5) |  |
| N Stage (%) | N0 | 66 (28.3) | 13 (21.3) | 53 (30.8) | 0.511 |
|  | N1 | 62 (26.6) | 17 (27.9) | 45 (26.2) |  |
|  | N2 | 52 (22.3) | 14 (23.0) | 38 (22.1) |  |
|  | N3 | 47 (20.2) | 16 (26.2) | 31 (18.0) |  |
|  | NX | 6 (2.6) | 1 (1.6) | 5 (2.9) |  |
| T Stage (%) | T1 | 10 (4.3) | 2 (3.3) | 8 (4.7) | 0.544 |
|  | T2 | 58 (24.9) | 13 (21.3) | 45 (26.2) |  |
|  | T3 | 109 (46.8) | 27 (44.3) | 82 (47.7) |  |
|  | T4 | 55 (23.6) | 19 (31.1) | 36 (20.9) |  |
|  | TX | 1 (0.4) | 0 (0.0) | 1 (0.6) |  |
| Person neoplasm cancer status (%) | NA | 21 (9.0) | 4 (6.6) | 17 (9.9) | 0.711 |
|  | Tumor Free | 159 (68.2) | 42 (68.9) | 117 (68.0) |  |
|  | With Tumor | 53 (22.7) | 15 (24.6) | 38 (22.1) |  |
| Primary lymph node presentation assessment (%) | NA | 6 (2.6) | 0 (0.0) | 6 (3.5) | 0.33 |
|  | No | 16 (6.9) | 4 (6.6) | 12 (7.0) |  |
|  | Yes | 211 (90.6) | 57 (93.4) | 154 (89.5) |  |
| Race (%) | NA | 18 (7.7) | 4 (6.6) | 14 (8.1) | 0.953 |
|  | Asian | 46 (19.7) | 13 (21.3) | 33 (19.2) |  |
|  | Black or African American | 10 (4.3) | 3 (4.9) | 7 (4.1) |  |
|  | White | 159 (68.2) | 41 (67.2) | 118 (68.6) |  |
| Radiation herapy (%) | NA | 10 (4.3) | 5 (8.2) | 5 (2.9) | 0.141 |
|  | No | 177 (76.0) | 42 (68.9) | 135 (78.5) |  |
|  | Yes | 46 (19.7) | 14 (23.0) | 32 (18.6) |  |
| In pancanpathways freeze (%) | No | 21 (9.0) | 5 (8.2) | 16 (9.3) | 1.000 |
|  | Yes | 212 (91.0) | 56 (91.8) | 156 (90.7) |  |

***P* value**: Wilcoxon test rank sum or Fisher’s exact test (two sided) was used for the comparison between the fusion burden high and low groups.
